# Homelessness among a cohort of women in street-based sex work: the need for safer environment interventions

**DOI:** 10.1186/1471-2458-11-643

**Published:** 2011-08-12

**Authors:** Putu Duff, Kathleen Deering, Kate Gibson, Mark Tyndall, Kate Shannon

**Affiliations:** 1 British Columbia Centre for Excellence in HIV/AIDS, St. Paul's Hospital, 608-1081 Burrard Street, Vancouver, BC, V6Z 1Y6, CANADA; 2 School of Population and Public Health, University of British Columbia, 5804 Fairview Avenue, Vancouver, BC, V6T 1Z3, CANADA; 3 WISH Drop-In Centre Society, Vancouver, BC, V6B 1S5, CANADA; 4 Department of Medicine, University of British Columbia, St. Paul's Hospital, 608-1081 Burrard Street, Vancouver, BC, V6Z 1Y6, CANADA

## Abstract

**Background:**

Drawing on data from a community-based prospective cohort study in Vancouver, Canada, we examined the prevalence and individual, interpersonal and work environment correlates of homelessness among 252 women in street-based sex work.

**Methods:**

Bivariate and multivariate logistic regression using generalized estimating equations (GEE) was used to examine the individual, interpersonal and work environment factors that were associated with homelessness among street-based sex workers.

**Results:**

Among 252 women, 43.3% reported homelessness over an 18-month follow-up period. In the multivariable GEE logistic regression analysis, younger age (adjusted odds ratio [aOR] = 0.93; 95%confidence interval [95%CI] 0.93-0.98), sexual violence by non-commercial partners (aOR = 2.14; 95%CI 1.06-4.34), servicing a higher number of clients (10+ per week vs < 10) (aOR = 1.68; 95%CI 1.05-2.69), intensive, daily crack use (aOR = 1.65; 95%CI 1.11-2.45), and servicing clients in public spaces (aOR = 1.52; CI 1.00-2.31) were independently associated with sleeping on the street.

**Conclusions:**

These findings indicate a critical need for safer environment interventions that mitigate the social and physical risks faced by homeless FSWs and increase access to safe, secure housing for women.

## Background

Emerging research suggests substantial health inequities exist among individuals without adequate, safe, and affordable shelter. Homelessness represents a unique social and physical environment that has been shown to substantially influence distribution of health inequities, risk taking and adverse health outcomes among marginalized populations [1,2]. "Absolute homelessness" is defined as "individuals living in the streets with no physical shelter of their own, including those who spend their nights in emergency shelters"[3]. Homelessness is a growing concern worldwide: according to a 2005 count, one billion people lack adequate housing, and approximately 100 million do not have housing at all [4]. In North America, homelessness is on the rise in many urban centres [5]. A 2005 homelessness count in the US estimated that 744,313 people experienced homelessness nationwide, with homelessness heavily concentrated in the country's major cities [5,6]. In the greater region of Vancouver, Canada, the total number of homeless individuals is increasing; a 2008 homelessness count identified 2,660 homeless people, over double the 2002 estimate [6]. The high rates of homelessness in the greater Vancouver region suggests that current poverty-alleviation and housing interventions are inadequate in curbing homelessness in the city [6].

Of particular importance, despite a large body of research examining the individual, social and physical contexts of homelessness among injection drug users (IDU) to date [1,7], there remains limited research documenting the prevelance and correlates of homelessness among street-based sex work populations, or how patterns of risk compare with their housed counterparts. Furthermore, the few studies to date among female sex workers (FSWs) have been cross-sectional. For example, in Miami, Florida, a recent cross-sectional study among street-based FSWs documented a higher number of vaginal and oral sexual transactions with clients, increased odds of engaging in unprotected vaginal intercourse and more frequent accounts of exchanging sex while high on drugs among homeless sex workers [8]. Clients of homeless FSWs were also more likely to refuse to use condoms compared to clients of more stably housed FSWs. This study provides important cross-sectional data on the sexual risks among homeless FSWs. Given that work environment factors have been increasingly shown to play a critical role in shaping health risks among FSWs, including negotiation of sexual risks, and violence [1,8,9], the context of absolute homelessness warrants further investigation.

An array of health problems have been associated with being homeless, including mental illness, physical violence, and substance abuse [2,7,9]. The convergence of these factors may elevate an individual's risk for homelessness, leading to the concept of "hard to house" individuals. Higher rates of drug use and sharing of needles have been observed among homeless compared to non-homeless individuals [1,10,11]. Having a greater number of sexual partners, engagement in unprotected sex and involvement in sex work have also been linked to homelessness and/or housing instability [12,13]. Many homeless persons are confronted with environmental conditions that may further exacerbate drug and sexual practices, placing them at higher risk for HIV infection. For example, homelessness and unstable housing have been associated with sharing injection drug paraphernalia (rigs, needles)[11] and the use of shooting galleries [14]. Persons who are homeless or unstably housed have been found to have HIV rates that are up to nine-fold higher than those who are stably housed [15]. In addition, evidence suggests that homeless persons experience numerous barriers to accessing health care and harm reduction services [16].

In order to address the dearth of longitudinal research on the individual, interpersonal and work environment factors associated with homelessness among female sex workers (FSWs), our study aimed to evaluate the prevalence and correlates of absolute homelessness (sleeping on the street) among a prospective cohort of street-based FSWs in Vancouver, Canada.

## Methods

Data were drawn from a community-based prospective cohort that has been described in detail previously [17]. Briefly, 252 street-based FSWs (response rate of 94%) were recruited and consented to participate in the study between 2006 and 2008. Based on the mapping of solicitation spaces ('sex work strolls'), a time-space sampling strategy was employed to recruit hard-to-reach populations by sampling at times and places where they often congregate. Unlike other sampling strategies, physical spaces instead of persons are the primary sampling unit [18]. The outreach team of current/former FSWs recruited participants at staggered working hours and locations at sex work strolls, using vehicles for late-night outreach for safety and increased coverage [17]. The study's eligibility criteria included being female or transgender aged 14 years or older, actively engaging in street-level sex work and using illicit drugs within the past month (excluding marijuana). This analysis was restricted to three visits over an 18 month period; participants completed baseline and at least one of two semi-annual follow up visits which consisted of an interview-administered questionnaire by a peer researcher (current/former street-based FSW), a nurse-administered pre-test counseling questionnaire, and HIV screening. Respondents received $25 honoraria compensation at each 6-monthly visit for their time and expertise. This research received ethical approved by UBC/Providence Health ethics review board.

### Dependent variable

Our dependent variable was 'absolute homelessness' in the previous 6 months based on a 'yes' response to the survey item "Have you slept on the street for one night or longer over the previous 6-month period?" Interviewers were trained to ensure that only true cases of homelessness were coded as positive responses.

### Explanatory variables

Individual, interpersonal and contextual/work environment factors were considered a priori based on our earlier qualitative research, and the homelessness literature. As previously [19], age was considered a continuous variable (years) and ethnicity was defined as Caucasian vs. non-Caucasian. Individual drug use patterns included daily cocaine and heroin injection, crystal methamphetamine use (injection/non-injection). As in our previous work [20], given the high rates of crack cocaine among street-based FSWs, we have stratified intensity of daily crack use at the median (10 or more rocks per day). Interpersonal variables of interest included servicing a higher number of clients per week (10+ vs less), inconsistent condom use by clients, being pressured into sex without a condom, having borrowed used syringes and pipes, and having experienced a 'bad date' (physical and/or sexual violence by a client), within the past 6 months. As homelessness has previously shown to be associated with sexual violence by non-commercial partners [20], we adjusted our model for this potential confounding effect. Work environment factors of interest included primary types of outdoor solicitation spaces (main streets/commercial corridors, alleys/industrial areas, residential communities), as well as servicing in outdoor public spaces (alleys, industrial settings) as compared to indoor spaces.

### Statistical analysis

Data was analyzed longitudinally. Baseline variables of age, ethnicity and education were considered as fixed covariates. All other factors were treated as time-updated covariates that referred to experiences occurring during the previous six-month period. As previously [21], data from each participant's baseline and follow up were included and analyzed using generalized estimating equations (GEE), which accounted for each individuals' repeated measurements over the 18-month observation period; thus, data from each participant's follow-up visit was included. These methods provided standard errors adjusted by repeated observations per person using an exchangeable correlation structure. Missing data were addressed through the GEE estimating mechanism, which uses the all available pairs method to encompass the missing data from dropouts or intermittent missing data. All non-missing pairs of data are used in the estimators of the working correlation parameters. Given the cyclical nature of homelessness, this method allowed us to analyze factors associated with the outcome of sleeping on the street in each 6-month period.

Descriptive statistics (e.g. prevalence, medians and interquartile range [IQR]) of baseline individual, interpersonal and work environment factors were presented, stratified by homelessness. Bivariate and multivariable logistic regression with GEEs was used to examine the relationship between individual, interpersonal and work environment factors and being homeless in the previous six months. Bivariate analyses were used to examine associations and test for potential collinearity or effect modification. P-values were generated using the Fisher's test of exact probability when one or more observations was less than or equal to five. A multivariate logistic regression model was constructed using GEE and subsequently fitted with factors that were significantly associated with homelessness at a p < 0.10-level to adjust for potential or known confounders. Variables were retained as significant in multivariable analyses at p < 0.05. The p-values reported are two-sided; bivariate and adjusted odds ratios (OR and aOR respectively) with 95% confident intervals (95%CIs) were reported.

## Results

This analysis was restricted to 252 sex workers who completed baseline and up to two follow-up surveys between 2006 and 2008. Just over half (51%) were Caucasian, and 49% were non-Caucasian (Indigenous/Aboriginal (including, being of First Nations, Metis, Inuit ancestry) or another visible minority). The median age of participants was 35 years [IQR: 25-41]. All participants self-identified as women, of whom sixteen participants (6.3%) were transgendered (male-to-female). The lifetime prevalence of absolute homelessness was 88%, with a median age of first sleeping on the street of 17 years [IQR: 14-25]. Over 18-months follow-up period, 43% of participants reported being homeless (sleeping on the street) at least once, suggesting that many of these women cycle in and out of homelessness.

The unadjusted and adjusted odds ratios in the multivariate analysis are presented in Tables 1 and 2. In bivariate analysis, injecting heroin (OR = 1.49; 95%CI 1.03-2.15), injecting or smoking crystal methamphetamine (OR = 2.21; 95%CI 1.26-3.87), injecting cocaine (OR = 1.17; 95%CI 0.80-1.69) and intensive crack use (OR = 1.65; 95%CI 1.19-2.30) within the past six months, having borrowed a used syringe/pipe (OR = 0.32; 95%CI 0.02-0.66), number of clients per week (OR = 1.69; 95%CI 1.12-2.54), sexual violence (OR = 1.99; 95%CI 1.03-3.83) and servicing clients in outdoor spaces (OR = 1.81; 95%CI 1.29-2.54) were all found to be significant. In the multivariate GEE logistic regression analyses, sexual violence by non-commercial partners (aOR = 2.14; 95%CI 1.06-4.35), servicing a higher volume of clients (10+ per week vs < 10) (aOR = 1.68; 95%CI 1.05-2.69), intensive, daily crack use (aOR = 1.65; 95%CI 1.11-2.45), servicing clients in public spaces (aOR = 1.52; 95% CI 1.00-2.31), and younger age (aOR = 0.93; 95%CI 0.93-0.98), were independently correlated with sleeping on the street.

**Table 1:** Sample characteristics for individual, interpersonal and sex work environment factors among homeless and housed street-based FSWs

Characteristic	Absolute Homeless FSWs (last 18 months) 43.32 (%) n = 107	Housed FSWs (last 18 months) 56.68 (%) n = 140	p - value
**Individual Sociodemographic Factors**			
Age (years, Interquartile range)	27 [ IQR^1^:23-37]	38 [IQR: 32-42]	< .001
Ethnicity			
Caucasian	54 (50.94)	82 (58.99)	
Aboriginal	52 (49.06)	57 (41.01)	0.571
Education			
Less than high school	75 (42.61)	101 (57.39)	
High School Graduate	22 (48.89)	23 (51.11)	0.967
College/University	9 (36.00)	16 (64.00)	0.292
*Drug Use Patterns*			
Cocaine injection *	35 (44.30)	44 (55.70)	0.042
Heroin injection*	59 (49.17)	61 (50.83)	0.033
Crystal methamphetamine*	22 (68.75)	10 (31.25)	0.005
Intensive, daily crack cocaine smoking)*	47 (48.45)	50 (51.55)	0.003
**Interpersonal Factors**			
Receptive sharing of used syringes/pipes*	75 (75.00)	60 (60.00)	0.064
Number of clients per week (10+)	40 (49.38)	41 (50.62)	0.012
Consistent condom use by clients*	16 (55.17)	13 (44.83)	0.957
Physical/sexual violence by client*	25 (47.17)	28 (52.83)	0.596
Physical violence by an intimate partner*	31 (47.69)	34 (52.31)	0.119
Sexual violence by an intimate partner*	5 (45.45)	6(54.55)	0.039
**Physical Work Environment Factors**			
Primarily solicits clients on main streets/commercial areas*	27 (81.82)	6 (18.18)	0.453
Primarily services clients in outdoor public spaces (streets, alleys, parks)*	43 (84.31)	8 (15.69)	0.103

^1 ^IQR = interquartile range

* = last 6 months.

**Table 2:** Unadjusted and adjusted odds ratios (ORs) and 95% confidence intervals (95%CI's) for the relationship between individual-level, interpersonal and sex work environment factors and homelessness among street-based FSWs in Vancouver

Characteristic	Unadjusted Odds Ratio (95% CI)	Adjusted Odds Ratio (95% CI)
**Individual Sociodemographic Factors**		
Age	0.94 (0.92-0.97)	0.95 (0.93-0.98)
Aboriginal† (vs. non-aboriginal)	1.14 (0.73-1.76)	
Education		
Less than high school completion	Reference	
High school graduate†	1.32 (0.55-3.15)	
Education, any college/university†	1.70 (0.62-4.64)	
**Drug Use Patterns**		
Cocaine Injection*†	1.17 (0.80-1.69)	
Heroin Injection*	1.49 (1.03-2.15)	1.16 (0.76-1.79)
Crystal methamphetamine use *	2.21 (1.26-3.87)	1.57 (0.91-2.71)
Intensive, Daily Crack Cocaine Smoking*	1.65 (1.19-2.30)	1.65 (1.11-2.45)
**Social/Interpersonal Factors**		
Receptive sharing of used syringe/pipe†	0.32 (0.02-0.66)	
Number of clients per week (10+)*	1.69 (1.12-2.54)	1.68 (1.05-2.69)
Consistent condom use by clients*†	1.01 (0.62-1.65)	
Physical/sexual violence by client *†	1.13 (0.72-1.79)	
Physical violence by intimate partner*†	1.34 (0.93-1.93)	
Sexual violence by intimate partner *	1.99 (1.03-3.83)	2.14 (1.06-4.35)
**Physical Work Environment Factors**		
Primarily solicits clients on main streets/commercial areas*†	1.18 (0.77-1.80)	
Primarily services clients in outdoor public spaces (streets, alleys, parks)*	1.81 (1.29-2.54)	1.52 (1.00-2.31)

* = last 6 months

†Variable not entered into logistic model.

## Discussion

This study is one of few that examines the prevalence and correlates of homelessness among street-based FSWs. The results demonstrate a staggering prevalence of both lifetime and recent homelessness among street-based FSWs, with a median age of first sleeping on the street during adolescence. Of particular concern, after adjusting for individual and interpersonal risks, homeless street-based FSWs were more likely to be younger, to experience sexual violence by non-commercial partners, to service a higher volume of weekly clients, to report intensive, daily crack smoking, and to exchange sex in outdoor spaces (as compared to indoor settings).

These findings collectively highlight the intersecting social and physical contexts of place in shaping health inequities among street-based FSWs. In our study, homeless street-based FSWs were 68% more likely to service a high number of clients (10+) per week compared to their housed counterparts, pointing to increased economic dependence on sex work for survival among impoverished women. This finding persisted even after adjustment for frequency and intensity of drug use, suggesting that lack of a basic necessity such as housing combined with the immediacy of sleeping on the street may confer additional need to exchange sex for basic resource needs, such as shelter or food. Our results extend earlier studies among homeless and marginally housed youth and IDU that found higher number of sexual partnerships than their more stably housed counterparts [10,12], perhaps through commercial sex exchanges. Similarly, Surratt and Inciardi (2004) found significantly more frequent vaginal and oral sex acts among homeless FSWs compared to their housed counterparts. Sexual violence by non-commercial partners is higher among homeless street-based FSWs suggesting that lack of access to safe, affordable spaces may reduce street-based FSWs' capacity to negotiate safety and elevate their risk for exploitation and abuse by intimate partners and other sexual partners. Other studies in North America have observed heightened risk of physical and sexual violence among homeless women [22]. Qualitative studies have highlighted that women immersed in the street economy occupy a subordinate role in the male-centred street ideology, and are often the victims of exploitation, physical and symbolic violence [23]. Street-entrenched women often enter intimate partnerships as a strategy for protection from structural, symbolic and interpersonal violence intrinsic to life on the street, however the power imbalances arising from these partnerships sometimes trap women in abusive relationships [23].

Importantly, contrary to a recent study among homeless and unstably housed male and female IDUs that found increased risk of unprotected sex compared to their stably housed counterparts [12], there were no differences in condom use among homeless and housed street-based FSWs. Instead, in our study, homeless street-based FSWs were more likely to work in public spaces, a context previously shown to be correlated with geographic 'hotspots' for increased coercive unprotected sex by clients in this setting [24]. These findings suggest that factors relating to unsafe sex work environments may be more important in the context of condom use negotiation and violence among street-based FSWs. However, the social and physical context of the lack of availability of safe places to sleep for street-based FSWs may play a more distal role on the causal pathway to unprotected sex by removing options to service clients indoors, within a setting where criminalization and enforcement are already displacing much of the street-based sex market to outlying areas.

Finally, unlike earlier investigations that have focused exclusively on IDUs, slightly less than half of our sample of street based FSWs were injectors while the majority smoked crack cocaine. In our study we found that being homeless was significantly associated with intensive, daily crack smoking. Our findings suggest that the pressures of living on the street may contribute to heightened levels of crack use among homeless street-based FSWs. Since many of the women in our sample live in Vancouver's downtown eastside, an area characterized by homelessness, poverty and high levels of drug use, this setting may increase street-based FSWs' exposure to high-risk environments such as crack houses, public drug markets, and shooting galleries that may elevate their crack consumption. Furthermore, living on the streets may also facilitate the creation of social ties with other drug users, encouraging and/or exacerbating intensive daily crack use among homeless street-based FSWs. Given that drug use is often an antecedent of homelessness and exchanging sex for survival [25], increased drug use among homeless street-based FSWs in our study was not unexpected. However, given the growing concern that crack cocaine smoking has emerged as a risk factor for HIV acquisition among IDUs in our setting [26], replacing cocaine injection in the earlier phases of the epidemic, our results have important public health implications. Further exploration of the contexts of homeless FSWs who smoke crack but do not inject is needed, combined with increased safer environment interventions targeting this population.

Collectively, our findings suggest that physical and social contexts of homelessness may contribute to or exacerbate violence, sexual- and drug- related risks and point towards the need for safer environment interventions that mitigate homelessness and associated risks. Safer environment interventions aimed at improving access and availability of safe, stable low-income housing for women in street-based sex work is particularly important in Vancouver, given the high costs of rental units and steady decreases in low income housing stock [27]. At the macro-level, policies that support expanding the continuum of safe, secure housing options for women are warranted, from low-threshold transitional shelters to supportive housing models. These housing options need to be coupled with higher rental subsidies and rental assistance programs that have proven effective elsewhere [28]. Furthermore, our results suggest that women- and sex work-only housing options need to be piloted and evaluated to reduce exposure to violence by intimate partners, strangers and mitigate sexual risks among street-based FSWs. These types of interventions should be supported by removal of criminal sanctions targeting sex work, given growing evidence of the links between enforcement of criminalized policies and displacement of street-based FSWs away from health and support services [9,24]. At the micro-level, other safer environment interventions that have proven effective in modifying the immediate risk environment and should be scaled up include peer-led outreach strategies [21]. Mobile health and support outreach services continue to be a critical, low-threshold model of connecting street-involved women with health and support services, and should be expanded to isolated sex work spaces.

This study has a number of limitations that should be noted. The findings from this study may not be generalizable to off-street sex workers (e.g. exotic dance, escort) or male sex workers. Given the observational nature of this research, we cannot determine causality, though some potential temporal bias may be reduced due to the use of generalized estimating equations that account for repeated responses by the same respondent. This study used self-report data, and women's responses may be subject to social desirability bias. However, a number of studies have found sex workers and drug users to provide truthful accounts of their sex and drug use activities when questioned in a non-threatening environment [29]. Due to a low prevalence of transgender women (6.3%), we were unable to tease out differences in homelessness by sexual identity in our current analysis. Finally, due to a recall period of 6 months, our results may be susceptible to recall bias. To reduce this bias, strategies such as using an individual event six months prior were used to facilitate recall. These results contribute to the growing body of literature advocating the importance of addressing environmental conditions that increase HIV risks, as a means to stemming the epidemic.

## Conclusion

In summary, this longitudinal study demonstrates a high prevalence of homelessness among street-based FSWs in an urban Canadian setting, with the median age of first sleeping on the street during adolescence. Of particular concern, 43% of women reported absolute homelessness over just 18-months of follow-up, suggesting women cycle in and out of housing. Homeless FSWs were younger, experienced higher exposure to violence by non-commercial partners, serviced a higher number of clients and were more likely to engage in sex work in public spaces as compared to their housed counterparts. Taken together, these findings support the need for safer environment interventions to modify the social and physical contexts of risk faced by homeless FSWs and increase access to safe, secure housing options for vulnerable women.

## Competing interests

The authors declare that they have no competing interests.

## Authors' contributions

KS had access to the data and takes full responsibility for the integrity of the data. PD and KS developed the analyses plan, and KD conducted the statistical analyses. PD wrote the first draft of the manuscript and integrated suggestions from all co-authors. All authors made significant contributions to the conception and design of the analyses, interpretation of the data, and drafting of the manuscript, and all authors approved the final manuscript.

## Pre-publication history

The pre-publication history for this paper can be accessed here:

http://www.biomedcentral.com/1471-2458/11/643/prepub
